# A Tendency Towards Details? Inconsistent Results on Auditory and Visual Local-To-Global Processing in Absolute Pitch Musicians

**DOI:** 10.3389/fpsyg.2019.00031

**Published:** 2019-01-22

**Authors:** Teresa Wenhart, Eckart Altenmüller

**Affiliations:** ^1^Institute of Music Physiology and Musicians’ Medicine, Hanover University of Music, Drama and Media, Hanover, Germany; ^2^Center for Systems Neuroscience, Hanover, Germany

**Keywords:** absolute pitch, cognitive style, week central coherence, autistic traits, musicians

## Abstract

Absolute pitch, the ability to name or produce a musical tone without a reference, is a rare ability which is often related to early musical training and genetic components. However, it remains a matter of debate why absolute pitch is relatively common in autism spectrum disorders and why absolute pitch possessors exhibit higher autistic traits. By definition absolute pitch is an ability that does not require the relation of tones but is based on a lower-level perceptual entity than relative pitch (involving relations between tones, intervals, and melodies). This study investigated whether a detail-oriented cognitive style, a concept borrowed from the autism literature (weak central coherence theory), might provide a framework to explain this joint occurrence. Two local-to-global experiments in vision (hierarchically constructed letters) and audition (hierarchically constructed melodies) as well as a pitch adjustment test measuring absolute pitch proficiency were conducted in 31 absolute pitch and 33 relative pitch professional musicians. Analyses revealed inconsistent group differences among reaction time, total of correct trials and speed-accuracy-composite-scores of experimental conditions (local vs. global, and congruent vs. incongruent stimuli). Furthermore, amounts of interference of global form on judgments of local elements and vice versa were calculated. Interestingly, reduced global-to-local interference in audition was associated with greater absolute pitch ability and in vision with higher autistic traits. Results are partially in line with the idea of a detail-oriented cognitive style in absolute pitch musicians. The inconsistency of the results might be due to limitations of global-to-local paradigms in measuring cognitive style and due to heterogeneity of absolute pitch possessors. In summary, this study provides further evidence for a multifaceted pattern of various and potentially interacting factors on the acquisition of absolute pitch.

## Introduction

Absolute pitch, the ability to name or produce a musical tone without any reference (Takeuchi and Hulse, 1993; Ward, 1999), has frequently been associated with autism (e.g., Heaton et al., 1998; Bonnel et al., 2003; for a review see Mottron et al., 2012) and autistic traits (Brown et al., 2003; Dohn et al., 2012). Ever since the association was first observed, a potential common framework for both phenomena and possible reasons for their joint occurrence have been matters of debate.

Absolute pitch is a rare condition (<1% in the general population, Profita et al., 1988) with a much higher incidence in professional musicians (up to 23%, Gregersen et al., 1999, 2001; Deutsch et al., 2006) and people with autism spectrum disorder (e.g., Heaton et al., 1998, 2008a,b; for a review see Mottron et al., 2012). In general, absolute pitch seems to be an excellent model to investigate the interaction of genetic and environmental influences on the acquisition and development of expert abilities (Zatorre, 2003). A large body of research exists on the heritability of absolute pitch (Baharloo et al., 1998; Gregersen et al., 1999; Athos et al., 2007), the importance of early musical training and sensitive periods (Gregersen et al., 2001; Russo et al., 2003; Schellenberg and Trehub, 2003; Deutsch et al., 2006) and neurophysiological and neuroanatomical differences related to absolute pitch (for a review see Zatorre, 2003; Bermudez and Zatorre, 2009) Among these, neuroanatomical research has demonstrated a larger left-right asymmetry of the planum temporale size in absolute pitch musicians (Schlaug et al., 1995), which seems to be based on a relatively smaller planum temporale in the right hemisphere (Keenan et al., 2001). Two-component frameworks (Zatorre, 2003; Levitin and Rogers, 2005) usually suggest two mechanisms that build up absolute pitch ability: early pitch categorization and pitch labeling. This has led to a range of studies investigating categorical perception (Siegel, 1974; Hedger et al., 2013), associative memory mechanisms of absolute pitch possessors (Elmer et al., 2013; Rogenmoser et al., 2015) and the relation of absolute pitch ability to speech (Deutsch et al., 2006; Deutsch et al., 2009; Oechslin et al., 2010). Interestingly, recent studies investigating brain structure and function of absolute pitch musicians have found a similar globally hypoconnected brain (e.g., Loui et al., 2011; Jäncke et al., 2012; Dohn et al., 2015) as is often seen in autism (e.g., Courchesne and Pierce, 2005; Supekar et al., 2013; Moseley et al., 2015) alongside with local hyperconnectivity (e.g., Loui et al., 2011; Supekar et al., 2013).

Autism is defined as a neurodevelopmental condition characterized by difficulties in social verbal and non-verbal communication, and by repetitive behaviors, restricted interests and sensory hyper- or hyposensitivities (Lai et al., 2013). Several authors have tried to explain absolute pitch with respect to autistic symptoms using theoretical concepts that describe a cognitive style with a tendency towards details. In autism literature, the weak central coherence account (Happé, 1999; Happé and Frith, 2006), the enhanced-perceptional functioning theory (Mottron et al., 2006, 2009), empathizing-systemizing-theory (Baron-Cohen, 2005, 2009) and the theory of veridical mapping (Mottron et al., 2012) are important frameworks, that include the concept of a detail-oriented cognitive style. At the same time, Chin (2003) has proposed that absolute pitch musicians may also share a tendency towards details, and that this may be associated with an early start in musical training before the age of seven. Chin (2003) argues that the more detailed view of the world that children exhibited up to the age of six (Poirel et al., 2008, 2011) leads to absolute pitch often only developing (or being maintained) during that period. Evidence for this view is provided by the doctoral thesis of Moreno Sala (2003). She also found that 5–7 year old children who scored higher on a test assessing sequential processing of information outperformed the other children on an absolute pitch task. Furthermore studies by Saffran and colleagues (Saffran and Griepentrog, 2001; Saffran, 2003) using statistical learning paradigms provide evidence that adults might be able to process absolute and relative cues while infants (8 months) rely heavily on absolute cues. This is suggestive of a potential shift in the influence of pitch and melody information on music perception – from the preference of absolute patterns towards a dominance of relative aspects of tones. While some of the neuroscientific results on absolute pitch have been discussed against the background of a possible relation between absolute pitch and autism (Loui et al., 2011, 2012; Jäncke et al., 2012; Dohn et al., 2015), cognitive style (in the sense of a tendency towards details) in absolute pitch musicians – as compared to the weak-perceptual-coherence account of autism – has never been investigated before.

Typically, paradigms to investigate detail vs. context-based cognition in autism follow the approach of the classical psychophysical experiments by Navon (1977) and consist of hierarchically organized visual (see e.g., Happé, 1999; Mottron et al., 2003; Happé and Frith, 2006; Bölte et al., 2007) or auditory (e.g., Mottron et al., 2000; Foxton et al., 2003; Justus and List, 2005; List et al., 2007; Bouvet et al., 2014) stimuli, e.g., a global letter shape consisting of small letters of either the same or another letter. A range of prior studies have provided evidence for a detail-oriented cognitive style in autistic people in vision (e.g., Grice et al., 2001; Mottron et al., 2003; Bölte et al., 2007; Pring et al., 2010; Russell-Smith et al., 2012). Recently, Bouvet et al. (2011) developed a paradigm to parallel the experiment in audition. Subjects had to rate the direction of short hierarchically-constructed melodies, where either the whole melody or parts of it were rising or falling. Again, people with autism spectrum disorders showed a detailed-oriented style in this auditory experiment on cognitive style (Bouvet et al., 2014). However, other authors were not able to replicate a detail-oriented performance of autistic people on hierarchical stimuli (e.g., Mottron et al., 1999, 2003; Foxton et al., 2003; Germain et al., 2018). It is currently under debate if this is caused by variations in experimental setup, variations among autistic people or failure of the theory. For this issue, we tried to reproduce the experiment of Bouvet et al. (2014) in such a manner, to be able to compare the results of our study on musicians with their results on autistic people and to exclude the confounding factor of varying the experimental setup. Construction of a visual global-local-experiment using Navon-letters will be paralleled accordingly.

If people with autism exhibit a more detail-oriented cognitive style, not only in vision but also in audition, this could be a possible reason for the high frequency of absolute pitch in autistic people, as absolute pitch – by definition – is an ability, that does not require the relation of tones but is based on a lower-level perceptual entity (single tones). Relative pitch by contrast always works by comparing two or more tones (higher level perceptual structure: intervals and melodies, i.e., relation between two or more pitches). However, it is unclear whether healthy absolute pitch possessors show a similar tendency for details in vision and or audition as autistic people, which could explain higher scores on autism self-rating scales. Prior studies have only investigated visuo-spatial abilities (Costa-Giomi et al., 2006), chord judgements (Ziv and Radin, 2014) and auditory digit span in absolute pitch possessors (Deutsch and Dooley, 2013) as well as the relation between relative and absolute pitch abilities in the same subjects (Ziv and Radin, 2014).

To our knowledge, this is the first study to investigate cognitive style in professional musicians with absolute vs. relative pitch, and its relation to accuracy of absolute pitch and autistic traits within the same sample. This study will therefore shed new light on the debate on why absolute pitch and autism are frequently associated and whether cognitive style could be their common framework.

## Materials and Methods

### Setting

The study was part of a larger project consisting of several experiments at the Institute of Music Physiology and Musicians Medicine of the University for Music, Drama and Media, Hannover. Two further experiments and EEG recordings were conducted within the same two sessions in the lab and are reported elsewhere (Wenhart et al., 2018a,b). For this reason, pitch adjustment assessment as well as cognitive tests from previous publications were also used as control variables here. Therefore, all subjects participated in three parts: an online survey and two appointments in the lab. The online survey was used for pitch identification screening and diagnostic as well as demographic questionnaires (see below). General intelligence tests, a musical ability test, a pitch adjustment test (Dohn et al., 2014) and two experiments assessing local-to-global processing both in vision and audition were conducted in the lab (see Table 1).

**Table 1:** Participants’ characteristics.

	AP (*n* = 25)			RP (*n* = 30)			*t*-test
	Mean	*SD*	Range	Mean	*SD*	Range	
Age	25.52	9.73	17–58	22.0	73.05	17–35	*t*(30.2) = −1.314; *p* = 0.197
SPM-IQ	111.7	15.82	73–132.25	113.65	13.47	86.5–134.5	*t*(47.4) = 0.485; *p* = 0.629
ZVT-IQ	122.62	13.2	101.5–145	120	13.4	97–143.5	*t*(51.5) = −0.728; *p* = 0.470
Hours main instrument	11271.2	8456.5	2190–39785	11476.82	13024.05	1606-58400	t(50.2) = 0.070; *p* = 0.944
AMMA	65.32	6.81	53–78	63.43	7.2	46–76	*t*(52.1) = −0.997; *p* = 0.324
MSI	206.48	17.2	161–232	210.07	15.1	185–246	*t*(48.4) = 0.821; *p* = 0.416
**PIS**	29.12	5.98	15–36	5.5	4.46	0–21^∗^	***t*(43.7) = −16.32; *p* = 2.2e-16**
**AQ**	21.12	6.26	12–36	16.7	5.66	6–27	***t*(49.0) = −2.722; *p* = 0.009**
**MAD**	35.05	21.82	9.8–93.33	288.99	84.73	91.04–467.52	***t*(33.5) = 15.798; *p* = 2.2e-16**
**SDfoM**	43.83	30.21	7.41–110.45	317.58	89.91	134.37–634.62	***t*(36.6) = 15.649; *p* = 2.2e-16**
Starting age	5.92	3.11	2–17	6.83	1.91	3–11	*t*(38.4) = 1.281; *p* = 0.208

*Age, nonverbal IQ (SPM), information processing capacity (ZVT), musical training (total hours during life span on main instrument), musicality (AMMA; MSI) and online pitch identification screening (PIS) for each group; ^∗^two RP reported not having absolute pitch but reached a screening score of 13 respectively 21. Because of this and their weak performance in the pitch adjustment test, the subjects were assigned to the RP group; Significant group differences are highlighted in bold. AQ refers to autism spectrum quotient (Baron-Cohen et al., 2001), MAD, Mean absolute derivation from standard tone; SDfoM, Standard deviation from own mean deviation.*

### Participants

In total, 31 absolute pitch (AP) musicians (16 female) and 33 relative pitch (RP) musicians (15 female) participated in the study. The above-mentioned online survey (UNIPARK software^1^) was used to recruit participants. They primarily were students or professional musicians at the University for Music, Drama and Media, Hanover; four AP and two RP were amateur musicians. As part of the online survey, a pitch identification screening test (PIS), consisting of 36 categorical, equal-tempered sine tones over a three octave range between C4 (261.63 Hz) and B6 (1975.5 Hz) was used to allocate the participants to groups (AP: >12/36 tones named correctly, else RP). There is currently no consensus about a cutoff in terms of percentage of tones named correctly to be defined as absolute pitch possessor. We chose a cutoff of 12/36 tones named correctly and not a higher, e.g., 50 or 80% cutoff, as some of our subjects reported to have absolute pitch (from our experience, professional musicians usually know whether they themselves have absolute pitch or not) and performed like absolute pitch possessors in the absolute pitch adjustment test in the lab [see section “Pitch Adjustment Test (PAT)”], despite their comparable weak performance in the pitch identification test online. The latter might have resulted from problems with tone presentation on some of the subjects’ personal audio-devices (reported during personal communication) or from uncontrolled experimental conditions (online study). To verify this decision, scatterplots visualizing the relation between pitch naming and adjustment have been inspected. Similarly, one participant yielding 21 correct in the online pitch naming test has been re-assigned to the RP group because of weak performance in the pitch adjustment test (and reporting not to have absolute pitch). Above chance performance of musicians without “real” absolute pitch is also occasionally seen in pitch naming tests, as professional musicians can have several experience based strategies for use in the test (e.g., having pitch of empty strings in string players or starting tones of famous melodies as comparison in mind), which they frequently reported to us. The lenient threshold of 12/36 therefore is due to technical issues and does not reflect a suitable cutoff for the assessment of other samples. However, the clear difference between AP and RP in pitch adjustment test, where RP strategies do not seem to help (personal reports after test), confirms the separation into the two groups in our sample (see Table 1). Non-native German speakers had the choice between a German and an English version of the experiments (four AP subjects). All participants but one reported no regular medication or drug intake. None of the participants reported any history of severe psychiatric or neurological condition. The AP group consisted of 15 pianists, nine string players, three woodwind instrumentalists, two singers and two brass players; the RP group consisted of 13 pianists, four string players, six woodwind instrumentalists, three bassists/guitarists/accordionists, three singers, one drummer, and three brass players. The Edinburgh Handedness Inventory (Oldfield, 1971) was used to assess handedness. Apart from one subject all AP were consistently right handed, whereas three RP were left-handed and two RP ambidextrous. This study was approved by the local Ethics Committee at the Medical University Hannover. All participants gave written consent.

Two standardized tests were used to assess general nonverbal intelligence and information processing speed: Raven’s Standard Progressive Matrices (Raven et al., 2004) and “Zahlen-Verbindungs-Test” (ZVT; Oswald, 2016). AMMA (Advanced Measures of Music Audiation; Gordon, 1989), Musical-Sophistication Index (GOLD-MSI; Müllensiefen et al., 2014) and estimated total hours of musical training within life span (internal online questionnaire) served to control for musical ability and musical experience.

### Experiments and Materials

#### Pitch Adjustment Test (PAT)

All participants performed two absolute pitch tests to assign them to groups AP or RP (pitch identification screening, online) and to assess the accuracy of absolute pitch under controlled conditions (pitch adjustment test, lab). During the pitch adjustment test (PAT; Dohn et al., 2014) participants have to adjust the frequency of a sine wave with random start frequency (220–880 Hz, 1 Hz steps) and try to hit a target musical note (letter presented centrally on PC screen, e.g., “F#/Gb”) as precisely as possible without the use of any kind of reference. Tones were presented through sound-isolating Shure 2-Way-In-ear Stereo Earphones (Shure SE425-CL, Shure Distribution GmbH, Eppingen, Germany) and participants were allowed to choose their octave of preference. The full test consisted of 108 target notes, presented in semi-random order in three blocks of 36 notes each (3 × 12 different notes per block) with breaks between the blocks. Online pitch modulation was provided by rotating a USB-Controller (Griffin PowerMate NA16029, Griffin Technology, 6001 Oak Canyon, Irvine, CA, United States). Participants could flexibly switch between rough and fine tuning by either turning the wheel (10 cent resolution) or by pressing it down while turning (1 cent resolution). Subjects were given a maximum of 15 s for each trial and could confirm their answer by pressing a button on a Cedrus Response Pad (Response Pad RB-844, Cedrus Corporation, San Pedro, CA, United States) to automatically proceed with the next trial. The final frequency at the time of the button press or at the end of the maximum time given was recorded. In both cases, the Inter Trial Interval (ITI) was set to 3000 ms. EEG was measured during the PAT but will be reported elsewhere. The final/selected frequencies in each trial were compared to the nearest target tone (<6 semitones/600 cent). The mean absolute deviation [MAD (1), (Dohn et al., 2014)] from the target tone is given as:

(1) $$\mathrm{MAD}=\frac{\sum_{\text{i}=1}^{{\text{N}}_{\text{adjustment}}}\left|C_{\text{i}}\right|}{N_{\text{adjustment}}},$$

This reflects the pitch adjustment accuracy of the participants and is calculated as the average of the absolute deviations *c*_i_ of the final/selected frequencies from the target tone (referenced to a 440 Hz equal tempered tuning). The consistency of the pitch adjustments (SDfoM, Standard deviation from own mean), possibly reflecting the tuning of the pitch template (Dohn et al., 2014), is then estimated by taking the standard deviation of the absolute deviations (2).

(2) $$\mathrm{SDfoM}=\frac{\sum_{\text{i}=1}^{{\text{N}}_{\text{adjustment}}}\left|C_{\text{i}}\right|}{N_{\text{adjustment}}-1}$$

Z-standardization of the MAD [Z_MAD, formula (3)] and SDfoM [Z_SDfoM, formula (4)] values relative to the mean and standard deviation of the non-AP-group were performed for statistical analyses, as originally proposed by Dohn et al. (2014).

(3) $$Z_{{\mathrm{MAD}}_{i}}=\frac{{\mathrm{MAD}}_{i}-\mu {\left(\mathrm{MAD}\right)}_{\mathrm{Non}-\mathrm{AP}}}{\sigma {\left(\mathrm{MAD}\right)}_{\mathrm{Non}-\mathrm{AP}}}$$

(4) $$Z_{{\mathrm{SDfoM}}_{i}}=\frac{{\mathrm{SDfoM}}_{i}-\mu \;{\left(\mathrm{SDfoM}\right)}_{\mathrm{Non}-\mathrm{AP}}}{\sigma \;{\left(\mathrm{SDfoM}\right)}_{\mathrm{Non}-\mathrm{AP}}}$$

#### Autistic Traits

The Adult Autism Spectrum Quotient (AQ, Baron-Cohen et al., 2001; German version by C.M. Freiburg^2^) was used to measure autistic traits. The questionnaire was presented within the online survey and consists of 50 items within five subscales (attention to detail, attention switching, imagination, social skills and communication). Items (half of them negatively poled) corresponding to either a mild or strong agreement with the autistic-like symptoms are given one point. The maximum AQ-Score therefore is 50.

#### Hierarchical Letters (HL)

**FIGURE 1:**
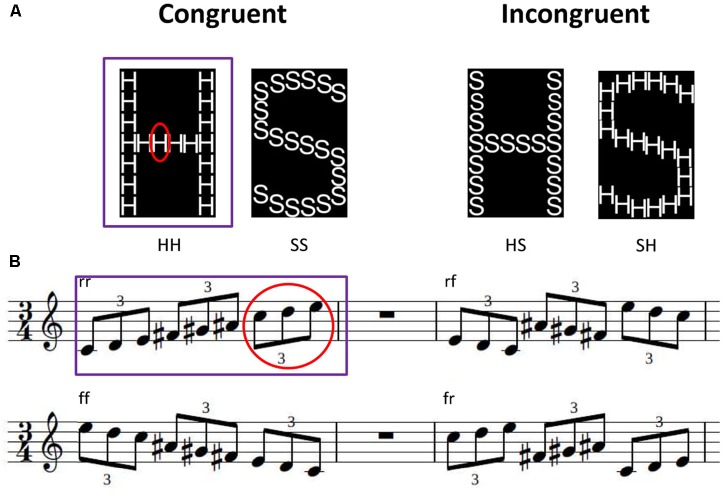
Examples of visual and auditory hierarchical stimuli of the Hierarchical letters **(A)** and Auditory global-local test **(B)**. Experiments are divided into two blocks, in which participants have to concentrate either on local elements (red; small letters or tone triplets) or on global shape (purple; big letter shape or whole melody). The resulting stimuli can be congruent (e.g., HH: big H, small H; rr = rising tones within whole melody and within triplets) or incongruent (e.g., SH: big S, small H; rf = rising tones within whole melody but falling within triplets). Melodic stimuli occur in different transpositions across all possible tonalities.

Four different hierarchical letters were constructed according to Navon (1977). The stimuli were either a global “H” or a global “S” each consisting either of small “H” or small “S” (see Figure 1A). Participants were asked to press a blue button for “H” and a yellow button for “S” or vice versa, depending on randomized allocation of the participants to the two experimental conditions (via the Cedrus Response Pad RB-844, Cedrus Corporation, San Pedro, CA, United States). Participants had to press the left button with the left hand, and the right button with the right hand. Order of the buttons (blue button left & yellow button right or vice versa) was randomized across participants and groups [see also section “Auditory Global-Local Test (AGLT)”]. All participants underwent two blocks of 80 trials each (20 for each stimulus condition). In one block, they were asked to press the two buttons according to the global level of the stimuli, in the other block according to the local level. The order of blocks was randomized across subjects, with half of AP and half of RP starting with the local, respectively global block. Each block had a self-timed break after trial 40. At the beginning of each block, four trials (one per condition) were presented for practice. Within each trial, a fixation cross was present at the center of the screen for 500 ms accompanied by a “beep” sound at the final 75 ms. Afterwards the stimulus was presented for 100 ms in one of the four quadrants around the center of the screen with a visual angle of 4.67° (viewing distance 60 cm; center of the images at [+−2.4, +−2.4] relative to screen center). A dotted mask appeared at the position of the stimulus for 1900 ms directly after the end of stimulus presentation, then followed by the next trial. The order of stimuli was randomized and stimulus positions were pseudo-randomized with the same stimulus never occurring twice in a row.

A total of nine participants (3 RP, 6AP) had to be excluded. They either misunderstood the experiment (*N* = 5) or were recognized as outliers during inspection of RT distributions across and within conditions (*N* = 4).

#### Auditory Global-Local Test (AGLT)

Hierarchical melodies were constructed according to Bouvet et al. (2014). Melodies consisted of nine tones in groups of three (triplets), lasted for 1900 ms (210 ms per note) and were presented through sound isolating Shure 2-Way In-ear Stereo Earphones (Shure SE425-CL, Shure Distribution GmbH, Eppingen, Germany). Melodies either successively ascended or descended in steps of two semitones, or the triplets ascended/descended and the next triplet started six semitones below respectively above the start of the prior triplet (see Figure 1B). Subjects were asked to judge either the direction of the melody as a whole, or the direction of the triplets in two different blocks of 80 trials each. Compared to Bouvet et al. (2014) we transposed the melodies to 11 different tonalities to avoid that subjects could use absolute pitch cues for the task. One of the transpositions (four trials, one of each condition) was taken for practice at the beginning of each block. The order of blocks (local vs. global condition) was randomized across subjects and groups. A break was given after the first half (40 trials) of each block. Subjects’ responses were recorded via the Cedrus Response Pad (Response Pad RB-844, Cedrus Corporation, San Pedro, CA, United States), with a right button press for ascending and a left button press for descending. Half of the participants of each group responded with the right button in yellow (and the left in blue) and half of the participants with the right button in blue (and the left in yellow). Color order of the buttons did not influence the results (*p* < 0.05 for all comparisons). Reaction times (RT) were calculated relative to the first tone, when a decision at the local, respectively, global level could be made (local: 2nd note, 210 ms; global: 4th note, 630 ms) to make reaction times between conditions comparable. During each trial, the word “attention” (German: “Achtung!”) was presented for 1000 ms at the center of the screen followed by the sound of the melody (1900 ms). Responses were allowed for a further 3100 ms after the end of the melody. After this time, or if a button press had occurred, the next trial followed after an ISI of 1000 ms.

A total of nine participants (4 RP, 5 AP) had to be excluded. They either misunderstood the experiment (*N* = 4) or were identified as outliers during inspection of RT distributions (see below) across and within conditions (*N* = 5).

### Statistical Analysis

All statistical analyses were conducted using the open-source statistical software package R (Version 3.5^3^) and the R packages car, effsize, psych, sjstats and stats. Regression plots of Figure 4 were created with the R package ggplot2.

All statistical analyses have been performed on three different dependent variables per experiment: total number of correct trials (correct), reaction time medians (RT), and a combined score “Speed-accuracy-composite-score” (SACS).

#### Collection and Preprocessing of Reaction Time (RT) Values

First, only reaction times for correctly answered trials in the experiments HL and AGLT were taken. RT distributions within and across subjects, conditions and groups were inspected. For AGLT, reaction times were calculated relative to the first possible time point of decision, i.e., 2nd note for local trials (RT-210 ms) and 4th note for global trials (RT-630 ms) to make RT’s comparable between conditions. Trials with physiologically impossible RT’s (i.e., < = 0) or extremely long RT’s (>1000 ms for HL) were then removed. In a next step, individual outliers defined as exceeding +/−2 times the mean absolute deviation from the median of each subject’s RT distribution were identified and the corresponding trials removed. The remaining trials were considered for further statistical group analysis using median and absolute deviation from median as dependent variables because of non-normality of the subjects’ individual RT distributions (the distribution of RT medians across subjects was normal). The process was performed separately for HL and AGLT trials.

#### Calculation of Speed-Accuracy Composite Scores (SACS)

Speed-accuracy-composite-score have been successfully used by Bouvet et al. (2014) for the auditory global-local paradigm, which served as a template for the present study (AGLT experiment). SACS combine the measures of accuracy (number of correct trials) and speed (reaction times, RT). Normalization (mean = 0, standard deviation = 1) of both scores was performed across all conditions (congruency, hierarchical level) and participants (groups). Equation 5 shows the calculation of SACS, which are given by the difference of *z*-values for accuracy (in % of correct trials) and *z*-values for reaction times (RT).

(5) SACS=Z(%)−Z(RT)

Therefore, SACS quantifies the performance in one score (e.g., correct) relative to the other (e.g., RT), so as to de-confound individual strategies – e.g., being fast but not very accurate or being very accurate at the expense of RT. A range of other studies, especially in the field of perception research, have made use of SACS and related scores (Austen and Enns, 2003; Collignon et al., 2010; Romei et al., 2011; Glaser et al., 2012).

#### Statistical Analysis of Group Differences

We expected group differences between AP and RP regarding performance on local versus global trials and an interaction between congruency and group for both, local and global trials. As Shapiro-Wilk-test did not reject the assumption of normality (*p* > 0.05 for all comparisons), parametric tests were used for statistical analysis. Three-way 2 × 2 × 2 ANOVAs with two within-subjects factors (congruency, hierarchical level) and one between-subjects factor (group) were performed for each experiment (HL and AGLT) on three dependent variables each: total number of correct trials (correct), reaction time medians (RT) and the combined score “Speed-accuracy-composite-score” (SACS). Additionally we performed 2 × 2 ANOVAs on SACS separately for local and global conditions of HL and AGLT, with between-subjects factor “group” and within-subjects factor “congruency.”

#### Statistical Analysis of Interference Effects

To investigate interference effects, i.e., either the interference of local elements on global perception or vice versa, and their correlation with autistic traits (AQ) and pitch adjustment accuracy (MAD, SDfoM), we calculated individual scores for global-to-local and local-to-global interference for RT, total number of correct trials and SACS according to Bouvet et al. (2011).Global-to-local interference (equation 6) is calculated as the difference between performance on local congruent (L_con_) minus local incongruent (L_inc_) trials (i.e., trails of the local experimental block), using RT, correct or SACS.

(6) $$\text{Global-to-local interference}={\text{L}}_{\text{con}}-{\text{L}}_{\text{inc}}$$

It reflects the interference of the unattended global level on the perception of the attended local level. Similarly, local-to-global interference takes global congruent (G_con_) minus global incongruent (G_inc_) trials (i.e., trials of the global experimental block)

(7) $$\text{Local-to-global interference}={\text{G}}_{\text{con}}-{\text{G}}_{\text{inc}},$$

and reflects the interference of the unattended local elements on the perception of the attended global level. Pearson’s product moment correlations were calculated to estimate the relationship between interference effects and autistic traits, respectively, absolute pitch performance.

## Results

With respect to general demographic aspects (age) and cognitive (SPM-IQ, ZVT-IQ) as well as musical abilities (AMMA, MSI, age of onset of musical training) no group differences were obtained (see Table 1). However, as expected, APs performed superior in pitch naming [PIS, t(43.7) = −16.32, p = 2.2e-16] and pitch adjustment [MAD: t(33.5) = 15.798, p = 2.2e-16; SDfoM: t(36.6) = 15.649, p = 2.2e-16]. Furthermore, APs on average exhibited higher autistic traits [AQ: t(49.0) = −2.722, p = 0.009]. As for correlations among the mentioned variables, pitch adjustment ability correlated not only with standard deviation from own pitch template in the pitch adjustment test (SDfoM: r = 0.939, p = 2.2e-16) and pitch naming (PIS: r = −0.859, p = 2.2e-16), but also (marginally) with age of onset of musical training (r = 0.262, p = 0.054) and autistic traits (AQ: r = −0.321, p = 0.017). Also standard deviation from own pitch template in the pitch adjustment test (SDfoM: r = −0.307, p = 0.023) and pitch naming (PIS: r = 0.391, p = 0.003) correlated with autistic traits.

### Auditory Processing

**FIGURE 2:**
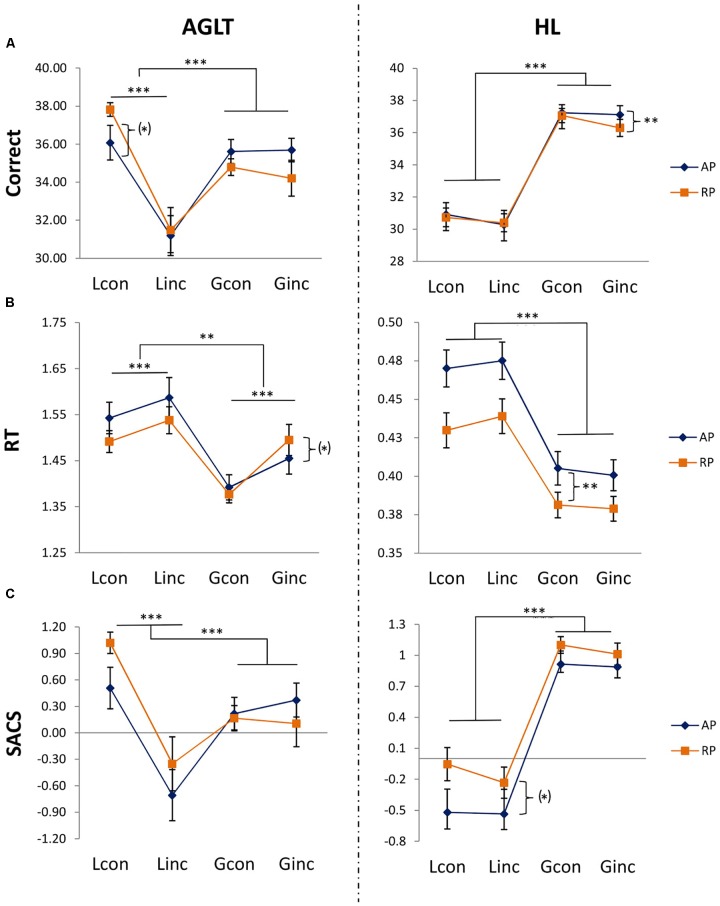
Differences on total of correct responses [row **(A)**], RT [row **(B)**] and speed accuracy composite score [SACS, row **(C)**] per condition (con = congruent, inc = incongruent; L = local, G = global) and group (AP = absolute pitch, RP = relative pitch). SACS: higher values indicate better performance. Bars represent standard errors. ^∗^*p* < 0.05. ^∗∗^*p* < 0.01. ^∗∗∗^ *p* < 0.001, (^∗^) *p* < 0.10.

Analyses revealed a main effect of hierarchical level for RT, *F*_RT_(1,53) = 45.33, *p* = 1.21e-08, $\eta_{p}^{2}$ = 0.75, [*F*_SACS_(1,53) = 0.17, *p* = 0.69; *F*_correct_(1,53) = 1.39, *p* = 0.24] and a main effect of congruency for all scores [*F*_RT_(1,53) = 34.65, *p* = 2.74e-07, $\eta_{p}^{2}$ = 0.55; *F*_SACS_(1,53) = 33.30, *p* = 4.19e-07, $\eta_{p}^{2}$ = 0.30; *F*_correct_(1,53) = 36.76, *p* = 1.44e-07, $\eta_{p}^{2}$ = 0.19]. Furthermore, there was a marginally significant main effect of group on RT [*F*_RT_(1,53) 3.33, *p* = 0.07, $\eta_{p}^{2}$ = 0.54]. Significant interactions for hierarchical level and congruency (see Figure 2) were also found for all scores [*F*_RT_(1,53) = 7.43, *p* < 0.009, $\eta_{p}^{2}$ = 0.12; *F*_SACS_(1,53) = 25.27, *p* = 6.05e-06], $\eta_{p}^{2}$ = 0.32; *F*_correct_(1,53) = 23.31, *p* = 1.21e-05, $\eta_{p}^{2}$ = 0.31], while a significant interaction of group and congruency was only found for total number of correct trials [*F*_correct_(1,53) = 4.21, *p* = 0.04, $\eta_{p}^{2}$ = 0.06] and marginally for SACS [*F*_SACS_(1,53) = 3.53, *p* = 0.07, $\eta_{p}^{2}$ = 0.04]. There were no three-way interactions. For means and standard deviations see Table 2.

**Table 2:** Auditory Processing (*N* = 55).

	Global processing		Local processing	
	Congruent	Incongruent	Congruent	Incongruent
**RT**				
AP	1.39 (0.14)	1.46 (0.17)	1.54 (0.17)	1.59 (0.22)
RP	1.38 (0.10)	1.49 (0.18)	1.49 (0.13)	1.54 (0.16)
**correct**				
AP	35.61 (3.24)	35.62 (5.97)	36.08 (4.65)	31.19 (5.34)
RP	34.79 (2.37)	34.79 (3.16)	37.83 (1.93)	31.48 (6.39)
**SACS**				
AP	0.22 (0.94)	0.37 (0.98)	0.51 (1.60)	−0.71 (2.21)
RP	0.17 (0.80)	0.10 (1.41)	1.02 (0.65)	−0.35 (1.65)

*Means (standard deviation) of auditory performance for total number of correct trials (correct,), reaction time (RT, ms) and speed-accuracy-composite-score (SACS) by group (absolute pitch, AP, vs. relative pitch, RP).*

**FIGURE 3:**
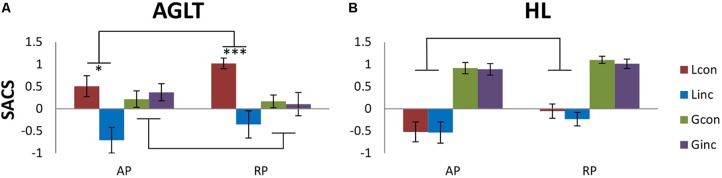
Speed accuracy composite score (SACS) for experimental conditions (hierarchical level, congruency) by group. **(A)** auditory processing (AGLT), **(B)** visual processing (HL). Marginal significant interaction between group and congruency for AGLT did not reach significance within local (*p* = 0.19) vs. global condition (*p* = 0.29). Higher values indicate better performance. HL similarly exhibited a weak tendency for a different effect of congruency within local condition (*p* = 0.19), but remained non-significant. Within-group differences for congruency are shown for all hierarchical levels and both experiments. ^∗^*p* < 0.05, ^∗∗^*p* < 0.01, ^∗∗∗^*p* < 0.001 (uncorrected).

The two-way ANOVA within the local condition revealed a main effect of congruency [*F*_congruency_(1,53) = 55.02, *p* = 9.61e-10, $\eta_{p}^{2}$ = 0.51] but not of group, nor was there any interaction [*F*_group_(1,53) = 1.58, *p* = 0.21, $\eta_{p}^{2}$ = 0.13; *F_congruency x group_* (1,53) = 1.72, *p* = 0.19, $\eta_{p}^{2}$ = 0.03]. The global condition yielded no significant main effects or interactions [*F*_group_(1,53) = 0.39, *p* = 0.53, $\eta_{p}^{2}$ = 0.01; *F*_congruency_(1,53) = 0.06, *p* = 0.81, $\eta_{p}^{2}$ < 0.01; F_*congruency x group*_ (1,53) = 1.13, *p* = 0.29, $\eta_{p}^{2}$ = 0.02]. Figure 3 shows differences between conditions per group and experiment. No *post hoc* tests were calculated as for huge amount of comparisons in our three 3-way-ANOVAS (inflation of alpha error) and the already inconsistent picture of main effects and interactions.

### Visual Processing

Analyses yielded main effects of hierarchical level for all scores [*F*_RT_(1,53) = 139.19, *p* = 2e-16, $\eta_{p}^{2}$ = 0.93, *F*_SACS_(1,53) = 94.85, *p* = 2.1e-13, $\eta_{p}^{2}$ = 0.93; *F*_correct_(1,53) = 119.69, *p* = 3.31e-15, $\eta_{p}^{2}$ = 0.89] and a marginal main effect of congruency for total number of correct trials [*F*_correct_(1,53) = 3.76, *p* = 0.06, $\eta_{p}^{2}$ = 0.04]. Significant interactions were found for hierarchical level and congruency (see Figure 2) on RT [*F*_RT_(1,53) = 5.56, *p* = 0.02, $\eta_{p}^{2}$ = 0.09], for group and hierarchical level on all scores [*F*_RT_(1,53) = 9.58, *p* = 0.003, $\eta_{p}^{2}$ = 0.49; *F*_SACS_(1,53) = 4.50, *p* = 0.04, $\eta_{p}^{2}$ = 0.39; *F*_correct_(1,53) = 6.01, *p* = 0.02, $\eta_{p}^{2}$ = 0.28), and marginally for group and congruency on RT [*F*_RT_(1,53) = 3.86, *p* = 0.05, $\eta_{p}^{2}$ = 0.05]. There were no three-way interactions. For means and standard deviations see Table 3.

**Table 3:** Visual Processing (*N* = 55).

	Global processing		Local processing	
	Congruent	Incongruent	Congruent	Incongruent
**RT**				
AP	0.41 (0.05)	0.40 (0.05)	0.47 (0.06)	0.48 (0.06)
RP	0.38 (0.05)	0.38 (0.04)	0.43 (0.06)	0.44 (0.06)
**correct**				
AP	37.24 (2.49)	37.12 (2.76)	30.92 (3.65)	30.28 (4.43)
RP	37.07 (2.43)	36.30 (2.91)	30.73 (3.19)	30.40 (3.05)
**SACS**				
AP	0.92 (0.64)	0.89 (0.64)	−0.52 (1.12)	−0.53 (1.19)
RP	1.10 (0.44)	1.01 (0.58)	−0.05 (0.88)	−0.23 (0.83)

*Means (standard deviation) of visual performance for total number of correct trials (correct), reaction time (RT, ms) and speed-accuracy-composite-score (SACS) by group (absolute pitch, AP, vs. relative pitch, RP).*

Two-way ANOVAs yielded a main effect of group [*F*_group_(1,53) = 3.98, *p* = 0.05, $\eta_{p}^{2}$ = 0.44] for the local condition [*F*_congruency_(1,53) = 1.79, *p* = 0.19, $\eta_{p}^{2}$ < 0.03; *F_congruency x group_* (1,53) = 0.33, *p* = 0.57, $\eta_{p}^{2}$ = 0.01] and no effects for the global condition [*F*_group_(1,53) = 0.18, *p* = 0.68, $\eta_{p}^{2}$ = 0.02; *F*_congruency_(1,53) = 0.84, *p* = 0.36, $\eta_{p}^{2}$ < 0.02; *F_congruency x group_* (1,53) = 0.62, *p* = 0.43, $\eta_{p}^{2}$ = 0.01]. No *post hoc* tests were calculated to avoid a huge amount of comparisons in our three 3-way-ANOVAS (inflation of alpha error) which would contribute to the already inconsistent picture of main effects and interactions.

### Interference Effects

In general, higher values for local-to-global interference or vice versa indicate higher interference by the local (respectively, global) level on incongruent trials. As smaller RT’s indicate better performance, RT interference effects are reversed (lower values indicating higher interference).

**FIGURE 4:**
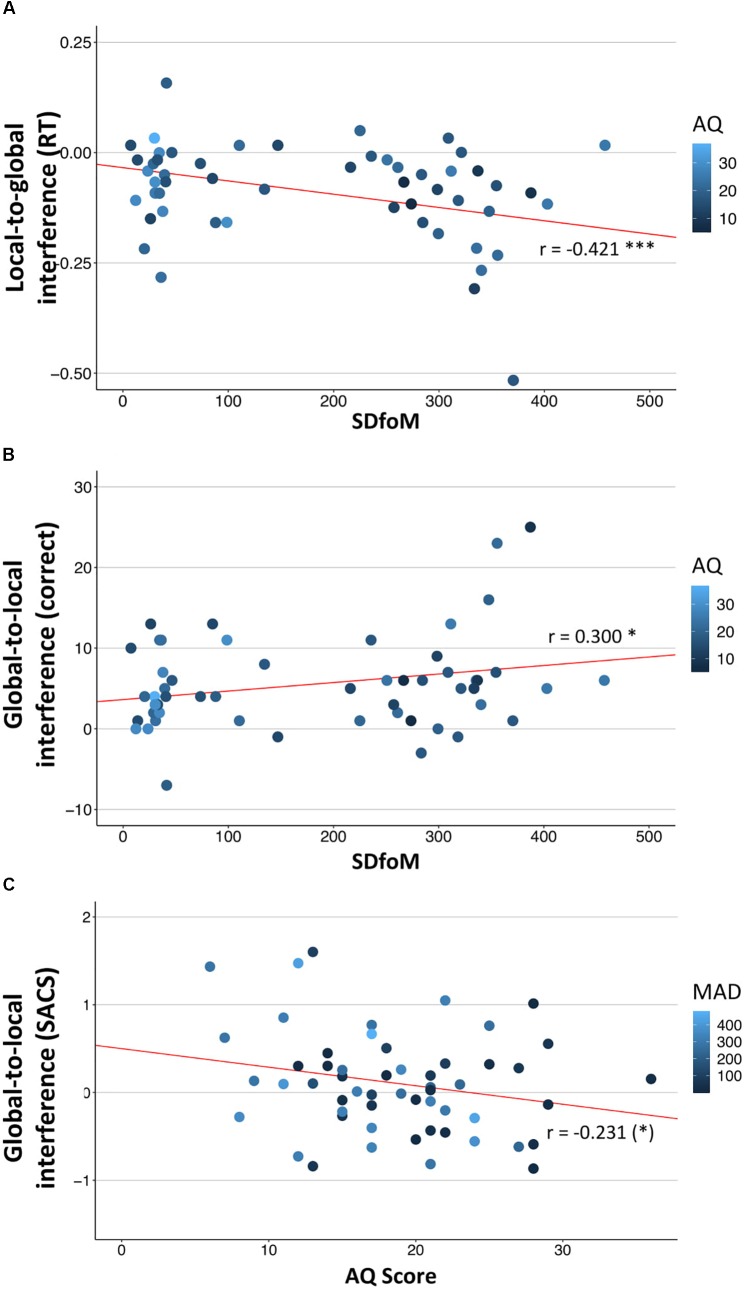
Correlations of visual and auditory interference with absolute pitch **(A,B)** and autistic traits **(C)** performance. **(A)** auditory local-to-global interference for RT (reaction times) correlates negatively with standard deviation from target tone in pitch adjustment test (*r* = −0.421); **(B)** auditory global-to-local interference for total number of correct trials (correct) correlates positively with standard deviation from target tone (*r* = 0.300); **(C)** Visual global-to-local interference (SACS,) correlates negatively with autistic traits (AQ-Score, *r* = −0.231, marginally significant). Higher values for interference (*y*-axis) indicate higher interference of the first named level (reverse for RT). Colors indicate values for pitch accuracy (MAD), respectively, autistic traits (AQ) and were only added for additional information (no significant bivariate correlations). Statistical values (pearson correlation coefficient *r*, significance indexes) correspond to the correlation between variables on *x* and *y* axis. ^∗^*p* < 0.10, ^∗^*p* < 0.05, ^∗∗^*p* < 0.01, ^∗∗∗^*p* < 0.001 (uncorrected).

Analysis of local-to-global interference revealed negative correlations between absolute pitch performance and RT local-to-global interference for the auditory domain (MAD: *r* = −0.295, *p* = 0.05; SDfoM: *r* = −0.421, *p* = 0.001). Therefore higher accuracy on absolute pitch tests (lower values MAD and SDfoM) is associated with weaker local-to-global interference in audition (see Figure 4). No local-to-global interference effects were found for the visual domain.

In the auditory domain, better performance (pitch template tuning, consistency) on absolute pitch tests (SDfoM) was furthermore correlated with reduced global-to-local interference in audition (correct: *r* = 0.300, *p* = 0.05; SACS: *r* = 0.242, *p* = 0.075, marginally significant). Higher autistic traits were associated with marginally lower global-to-local interference in the visual domain (*r* = −0.231, *p* = 0.090). However, all other correlations remained non-significant (see Tables 4, 5). Furthermore RTs did not correlate with pitch accuracy among AP musicians for congruent AGLT.

**Table 4:** Local-to-global interference (G_con_-G_inc_).

	AQ	MAD	SdfoM
**RT**			
AGLT	0.034 (0.806)	**−0.295 (0.029)**	**−0.421 (0.001)**
HL	0.019 (0.893)	−0.037 (0.789)	−0.033 (0.810)
**correct**			
AGLT	0.172 (0.210)	0.082 (0.550)	0.096 (0.486)
HL	−0.076 (0.583)	−0.024 (0.860)	0.023 (0.869)
**SACS**			
AGLT	0.141 (0.304)	0.105 (0.447)	0.117 (0.394)
HL	−0.038 (0.7845)	−0.064 (0.643)	−0.054 (0.696)

*Pearson’s product moment correlations and p-values for differences between global congruent (G_con_) and global incongruent (G_inc_) trials, separately for reaction time (RT, ms), total number of correct trials (correct) and speed-accuracy-composite-score (SACS). Correlations were calculated with autism traits (AQ) and absolute pitch accuracy (MAD, SDfoM). Significant values are highlighted in bold.*

**Table 5:** Global-to-local interference (L_con_-L_inc_).

	AQ	MAD	SdfoM
**RT**			
AGLT	0.202 (0.139)	0.030 (0.827)	−0.006 (0.965)
HL	0.171 (0.213)	−0.013 (0.927)	−0.022 (0.875)
**correct**			
AGLT	−0.126 (0.359)	0.184 (0.178)	**0.300 (0.027)**
HL	−0.161 (0.240)	−0.014 (0.919)	−0.114 (0.409)
**SACS**			
AGLT	−0.171 (0.211)	0.109 (0.429)	**0.242 (0.075)**
HL	**−0.231 (0.090)**	0.094 (0.495)	0.018 (0.898)

*Pearson’s product moment correlations and p-values for differences between global congruent (L_con_) and global incongruent (L_inc_) trials, separately for reaction time (RT, s), total number of correct trials (correct), and speed-accuracy-composite-score (SACS). Correlations were calculated with autism traits (AQ) and absolute pitch accuracy (MAD, SDfoM). Significant values are highlighted in bold.*

## Discussion

The present study is the first to investigate cognitive style, i.e., the tendency towards either the details or the global shape/context of sensory stimuli, in AP and RP musicians. Taken together, our results cannot confirm or reject the hypothesis that AP musicians have a more detail-oriented cognitive style compared to RP. The evidence is too weak and inconsistent across experiments and conditions to explain differences in AP performance based on cognitive style alone.

### Pitch Perception and Cognitive Style

Performance on auditory or visual hierarchically-constructed stimuli frequently used to assess cognitive style (Navon, 1977; Bouvet et al., 2011) did not reveal strong group differences between AP and RP musicians (see Figures 2, 3). As expected, global as well as congruent stimuli revealed a processing advantage both in terms of speed (reaction times) as well as accuracy (correct, speed-accuracy composite scores) independent of group. In general, AP and RP were similar in the degree of performance difference between local and global congruent and incongruent trials (three-way interaction). If anything, the groups might differ in performance on congruent vs. incongruent trials independent of hierarchical level, or vice versa. Reaction times in both audition and vision furthermore showed a tendency for slower responses of AP independent of experimental conditions (congruent vs. incongruent stimuli), which was especially prevalent in the local condition of the visual experiment. Interestingly, groups did not differ in basic information processing speed measured as a confounding variable by ZVT (“Zahlen-Verbindungs-Test”; Oswald, 2016), so general information processing ability cannot account for these differences. Possibly, absolute pitch possessors might be slower in judging relative information among tones (intervals, melodies) because of their automaticity of tone labeling, which has also been described with respect to interval labeling especially of out-of-tune reference notes in some (Miyazaki, 1995), but not all absolute pitch possessors (Dooley and Deutsch, 2011). However, this interpretation cannot explain slower reaction times in the vision. In summary, while frequent effects of congruency and local vs. global condition were independent of groups, there was no consistent tendency towards an advantage for particular processing levels (local vs. global congruent vs. incongruent trials) for the two groups, which would have been reflected in three-way interactions.

Correlation analysis revealed higher interference of local percept on global performance (lower local-to-global interference, see Figure 4A) is associated with higher accuracy in pitch adjustment test, but only for RT and only in audition. As we were expecting more detail-oriented perception for AP possessors (Chin, 2003; Mottron et al., 2012), this result actually stands against our hypothesis, as here RP are more affected by details in perceiving a global auditory percept. However, this was only present for RT measures, which alone might not comprise clear evidence in our experiments. Musical stimuli by their nature unfold over time and participants’ response latencies might differ according to their listening strategy. For example some individuals may listen to the whole stimulus, before deciding whether global or local changes were presented, whereas others may choose to press the button as soon as the crucial 4th tone is played (which allows them to notice the difference between global and local stimuli). In line with our hypothesis, reduced global-to-local interference in audition (total number of correct trials, speed-accuracy composite scores) is correlated with higher AP accuracy (see Figure 4B). In vision, however, higher autistic traits are associated with lower global-to-local interference [speed accuracy composite scores, see Figure 4C)]. Therefore, in audition, people who have a more accurate AP are less affected by the global shape when concentrating on local details, as are people with more autistic traits (in the same sample) in vision. However, we have to admit that this is a weak relationship as it is selective for certain performance measures and sensory domains. In contrast, prior research has shown that cognitive style is quite similar within subjects across audition and vision (Justus and List, 2005; Sanders and Poeppel, 2007; Bouvet et al., 2011). A possible explanation could be that our sample only consists of professional musicians and students at music universities. This is a highly auditorily trained population, a fact which might increase the likelihood of obtaining differing effects in audition and vision. Difference in audition might be caused by the differences in specific musical abilities (absolute pitch) whereas differences in vision maybe more domain general effects reflecting autism relevant mechanisms. We also have to draw attention to the problem of the confounding nature of absolute pitch and autistic traits, which was also present in our sample (see Table 1). Group differences and correlations with absolute pitch ability can therefore always also in part be confounded by autistic traits and vice versa. Other samples (e.g., autistic subjects with and without absolute pitch) would be necessary to analyze the reciprocal influence of those two. As for additionally controlling for musicality, this might be a very difficult task. Further limitations of our study are the absence of a non-musical control group as well as of a direct comparison to an autistic sample. However, as we tried to keep precisely with the experimental setup of Bouvet et al. (2014), we argue, that our results at least of the auditory experiments should technically be comparable to the autistic sample of Bouvet et al. (2014), apart from differences in musicality. In general, inconsistent and weak effects might also be due to subgroups within AP musicians, whereby not all AP musicians might exhibit heightened autistic traits and/or a detailed cognitive style. This view receives support from a range of research on AP showing various influences on the acquisition of the ability, including genetics (Baharloo et al., 1998; Gregersen et al., 1999, 2001, 2013), an early start of musical training (Baharloo et al., 1998; Chin, 2003; Gregersen et al., 2001; Bermudez and Zatorre, 2009; Gervain et al., 2013), a sensitive period (Saffran and Griepentrog, 2001; Saffran, 2003), musical education method (Gregersen et al., 2001) and nationality or mother tongue (Deutsch et al., 2006; Deutsch et al., 2009). However, larger sample sizes are needed to uncover subgroups in such a heterogeneous population.

### Hierarchical Stimuli and Cognitive Style

Despite the popularity of the weak-central-coherence account (Happé, 1999; Happé and Frith, 2006) and similar theories of autism (Baron-Cohen, 2009; Mottron et al., 2012, 2006) as well as of the global-local paradigms (Navon, 1977), a few authors have already raised criticism concerning these hypothetical concepts. First, global-local paradigms in the sense of Navon (1977), exhibit a huge variability of results across previous studies even in healthy populations. For example, various studies yielded heterogeneous results that might be caused by the relative size and the number of local elements used to construct the hierarchical stimuli in the respective studies (Kimchi and Palmer, 1982). Kimchi (1992) further emphasizes that global-local paradigms using hierarchically constructed stimuli might not even measure the degree of holistic perception, as being holistic (i.e,. properties that depend on the interrelations between component parts) is not necessarily the same as involving global precedence (i.e., processing of the higher level preceding that of the lower one). Therefore, not all global-to-local paradigms might be adequate to measure holistic perception in terms of Gestalt principles (Wertheimer, 1925). Furthermore, even evidence on a reduced global precedence effect as a result of a more detail-oriented perception in autism is contradictory (Ozonoff et al., 1994; Mottron et al., 1999, 2000, 2003; Foxton et al., 2003; Germain et al., 2018).

### Future Directions

Future studies should therefore address holistic vs. detailed perception using adapted paradigms (e.g., Kimchi, 1992; List et al., 2007; Sanders and Poeppel, 2007) to overcome restrictions of classical global-to-local paradigms (Navon, 1977). Furthermore, a consideration of neurophysiological or – anatomical correlates, especially asymmetries in hemispherical contributions, promises to offer a new contribution to the debate of detail-oriented processing style of AP musicians. Seminal work by Peretz and Morais (1987) and Peretz (1990), on patients with unilateral brain lesions (Peretz, 1990) and healthy non-musicians (Peretz and Morais, 1987) has shown a processing bias of local information by the left and global by the right hemisphere. This is especially interesting, as research from both fields, autism and absolute pitch, often reveals hemispherical associations (e.g., Keenan et al., 2001; Hyde et al., 2008; Brancucci et al., 2009; Wilson et al., 2009; Wengenroth et al., 2014; Dohn et al., 2015; Floris et al., 2016).

To sum up, the correlation analysis of global-to-local interference effects in particular revealed results in accordance with the hypothesis of a more detailed-oriented cognitive style in AP possessors, which is also associated with autistic traits within our sample. However, the inconsistency of the results – and the dissociation of a correlation of AP accuracy with auditory performance versus autistic traits with visual performance – remains to be understood.

## Data Availability Statement

The datasets generated and/or analyzed during the current study are not publicly available due to specifications on data availability within the ethics approval. Data are, however, available from the corresponding author upon reasonable request and with permission of the ethics committee of the Hanover Medical School.

## Ethics Statement

This study was carried out in accordance with the recommendations of the ethic committee of the Hanover Medical School (DE9515) with written informed consent from all subjects. All subjects gave written informed consent in accordance with the Declaration of Helsinki. The protocol (Approval no. 7372) was approved by the ethic committee of the Hanover Medical School (DE 9515).

## Author Contributions

TW designed the study, collected, analyzed, and interpreted the data. EA contributed to the design of the study and interpretation of the data. Both the authors read, improved, and approved the final manuscript.

## Conflict of Interest Statement

The authors declare that the research was conducted in the absence of any commercial or financial relationships that could be construed as a potential conflict of interest.

## Footnotes

1 https://www.unipark.com/

2 https://www.autismresearchcentre.com/arc_tests

3 https://www.r-project.org/
